# Impact of Gastroparesis on Outcomes After Pancreas Transplantation

**DOI:** 10.1097/TXD.0000000000001788

**Published:** 2025-04-09

**Authors:** Jonathan A. Fridell, Jeanne M. Chen, Emily A. Kerby, William A. Marshall, Andrew J. Lutz, John A. Powelson, Richard S. Mangus

**Affiliations:** 1 Department of Surgery, Indiana University School of Medicine, Indianapolis, IN.; 2 Department of Pharmacy, Indiana University Health, Indianapolis, IN.; 3 Department of Surgery, Henry Ford health, Detroit, MI.; 4 Department of Surgery, University of Colorado, Anschutz Medical Campus, Aurora, CO.

## Abstract

**Background.:**

Gastroparesis (GP) is a chronic disorder of the stomach characterized by delayed gastric emptying and frequently associated with longstanding diabetes. This is a single-center retrospective analysis designed to establish the prevalence and assess the impact on posttransplant outcomes of GP among pancreas transplant recipients.

**Methods.:**

Medical records for all recipients of pancreas transplants performed between January 2003 and December 2023 were reviewed. GP was defined by abnormal gastric-emptying scintigraphy or other motility study or a history of symptoms. Primary outcomes included graft loss and patient death. Clinical outcomes included length of stay after transplant and readmissions, including specifically for GP symptoms.

**Results.:**

Of 731 recipients, 156 (21%) were diagnosed with GP before transplant. Patients with GP were younger and more likely to be female individuals. Posttransplant, there was no difference in length of stay, graft survival, or patient survival. Patients with GP were more likely to be readmitted and to be specifically admitted for GP symptoms. Requirement for interventions was more common in patients with GP.

**Conclusions.:**

GP is identified with increased frequency among the specific patient population referred for pancreas transplant, and although it does not seem to affect allograft or patient survival, it does seem to have an impact on readmissions and the need for interventions.

Gastroparesis (GP) is a chronic disorder of the stomach characterized by a delay in gastric emptying without an identifiable mechanical obstruction.^1^ GP is categorized by its origin, with most being either related to diabetes mellitus (DM) or idiopathic, with a lesser number related to gastric surgery or a post–viral syndrome.^2^ The primary symptoms of GP include nausea, emesis, early satiety, anorexia, abdominal pain, and bloating.^1,3,4^ Epidemiologically, GP affects <0.1% of the population (24 per 100 000 persons) in the United States.^5^ More than 80% of affected patients with GP are female individuals. There is a prevalence of GP among patients with type 1 and 2 DM of 5% and 1%, respectively.^6,7^ However, chronic abdominal symptoms similar to GP have been described in up to 20% of patients with DM.^8,9^ It appears that GP associated with DM appears most commonly in patients who have a >10 y history of diabetes and is often accompanied by neuropathy, retinopathy, and nephropathy. Although the prevalence of GP is higher in patients with type 1 DM, there are many more patients with type 2 DM and with GP because of the much higher incidence of type 2 diabetes in the general population.^10^ Patients with DM who develop GP have lower health-related quality of life compared with those without GP.^11^ GP-related hospitalizations in the United States account for a growing proportion of healthcare dollars because the incidence of GP is increasing with the aging and more obese population at higher risk for diabetes.^12,13^

GP responds poorly to most medical therapies, primarily a combination of promotility and antiemetic agents, and symptoms often persist for years. Surgical therapies, including gastrectomy, intrapyloric botulinum toxin, gastric peroral endoscopic pyloromyotomy (G-POEM), and gastric electrical stimulation, have been described, with mixed results.^14-16^ The pathophysiology of GP is multifactorial and includes autonomic neuropathy, as well as myogenic mechanisms. In patients with DM, chronic oxidative stress leads to a loss of the interstitial cells of Cajal, which results in poor gastric peristalsis and atrophy of gastric smooth muscle.^17^ It has been suggested that improved glycemic control leads to improvement in autonomic dysfunction in patients with DM.^18,19^ However, there has been no evidence to suggest that GP improves with a complete resolution of diabetes, such as occurs with successful pancreas transplantation. The impact of pancreas transplantation on posttransplant GP and the impact of GP on post–pancreas transplant outcomes is unknown. Remarkably, considering the prevalence of GP in the population of candidates for pancreas transplantation who all have diabetes and the impact on the postoperative course of recipients, very little has been published on this chronic disease process in the pancreas transplantation literature. A single study of 42 simultaneous pancreas and kidney (SPK) transplant patients, published in 2004, found a lessening of gastrointestinal symptoms after transplant, with an increase in prescribed prokinetic and antisecretory medications.^20^ The present study reviews the clinical outcomes for 762 consecutive pancreas transplants from 2003 to 2023. This study seeks to establish the prevalence of GP in this specific population, define management strategies, and attempt to assess the impact of GP on posttransplant outcomes. Pre- and posttransplant use of promotility and antiemetic agents is reported.

## MATERIALS AND METHODS

The medical records for all adult, deceased donor pancreas transplants performed at Indiana University between January 2003 and December 2023 were reviewed (n = 762). Data were extracted from the comprehensive transplant recipient registry maintained at our center, individual written and electronic medical records, and the original donor and recipient medical histories. Inclusion criteria for this analysis included all transplant recipients undergoing either SPK transplant, pancreas after kidney (PAK) transplant, or pancreas transplant alone (PTA). For patients who underwent retransplantation, only the first transplant was included in the analysis so that each recipient was only included a single time in the data set. Pancreas transplants performed in conjunction with other organs, such as multivisceral transplant, lung/pancreas, or liver/pancreas, were excluded from this analysis. This resulted in 731 unique patients in the research cohort used for analysis. All recipients were listed for transplantation at Indiana University according to standard procedures and protocols as established by our center. Briefly, all patients had insulin-dependent diabetes. For those receiving a kidney transplant, candidates would need to meet listing and waiting time requirements according to Organ Procurement and Transplantation Network policy. PTA was indicated for potentially life-threatening complications of diabetes, particularly hypoglycemia unawareness. Our program does not have a strict age or body mass index (BMI) cutoff for evaluation or listing. Decisions are made on a case-by-case basis. For candidates that phenotypically have type 2 diabetes, our guidelines include insulin requirements of <100 units/d of insulin, and although there is no strict cutoff, we prefer a BMI of <30 kg/m^2^.

GP was defined as an abnormal finding on gastric-emptying scintigraphy, other abnormal motility study, or a documented history of symptoms consistent with gastroparesis for >3 mo.^21-24^ Neither the presence of abnormal scintigraphy nor a history of severe GP in isolation had any influence on decisions regarding listing of patients for transplant.

Primary transplant outcomes included early and late graft loss and patient death (90 d, 1 y, and 5 y). A 10-y Cox regression analysis was performed to compare long-term graft and patient survival for patients with and without GP while controlling for relevant covariates. Clinical outcomes included length of hospital stay, readmissions within 3 mo and 1 y, and readmissions with symptoms specific to gastroparesis. A detailed chart review was performed for each graft loss or patient death and is reported as a subgroup analysis. Pancreas allograft failure was defined by dependence on subcutaneous insulin administration. All occurrences and causes of graft loss or patient death were included in the final analysis. Additional data collected and analyzed with relevance to GP included serum albumin at the time of transplant and at 1 y posttransplant, change in BMI from transplant to 1 y posttransplant, both admission and discharge promotility agents and antiemetics, pre- and posttransplant need for esophagogastroscopy, gastric stimulator, and pyloric injection with botulinum toxin.

Demographic data were compared using standard chi-square and ANOVA testing for categorical and continuous variables, respectively. Kaplan-Meier analysis was used to calculate estimated months of graft and patient survival posttransplant. Retrospective collection, review, and analysis of data from the transplant center database was approved by the institutional review board of the Indiana University School of Medicine.

Pancreas allografts were procured after aortic flush with preservation solution and topical cooling with saline slush as previously described.^25^ The recipient operation was performed through a midline incision. The pancreas was routinely positioned with the tail toward the pelvis and the head and duodenum oriented superiorly to facilitate enteric anastomosis. Systemic venous drainage was performed to the vena cava. Arterial perfusion of the allograft was routinely established from the right common iliac artery, although on rare occasions, the inflow would be established either from the aorta or the left common iliac artery. All SPK transplants were performed with ipsilateral placement of both the kidney and the pancreas to the right iliac vessels as previously described.^26^ Pulsatile perfusion was used routinely for the renal allograft portion of the SPK transplant, regardless of the preservation solution used for organ procurement.^27^ All pancreas allografts were drained enterically using a stapled technique as described elsewhere.^28^

The induction immunosuppression protocol for SPK and PAK recipients consists of 5 doses of rabbit antithymocyte globulin (rATG) (1 mg/kg/dose) and maintenance with tacrolimus (target trough level of 8–10 ng/mL) and sirolimus (target trough level of 3–6 ng/mL). Steroids were exclusively used as a premedication for rATG and were discontinued after induction in all recipients, including PAK recipients receiving long-term steroids for remote renal transplant.^29^ PTA recipients received induction with rATG as described earlier and maintenance, including tacrolimus (target trough 6–8 ng/mL), sirolimus (target trough 3–6 ng/mL), and mycophenolate mofetil (500 mg po bid) or mycophenolic acid (360 mg po bid). If there were concerns regarding the absorption of tacrolimus due to vomiting, a sublingual formulation would be used instead of oral. In cases where the entire maintenance immunosuppression regimen was not tolerated well due to gastrointestinal issues in the setting of GP, particularly for the mycophenolate mofetil or mycophenolic acid as a third agent for PTA, azathioprine could be substituted. If the issues persisted, azathioprine was replaced with a monthly intravenous infusion of basiliximab (40 mg).^30^ All recipients received routine perioperative antibiotics, prophylaxis against cytomegalovirus with oral valgancyclovir, and prophylaxis against *Pneumocystis jiroveci* pneumonia with trimethoprim-sulfamethoxazole (Septra) unless contraindicated. Systemic anticoagulation was not routinely used unless the patient had a specific history of a coagulation disorder, although standard deep venous thrombosis prophylaxis and aspirin were initiated in all recipients after transplant.

In recognition of issues with gastroparesis specific to this population that were leading to prolonged length of hospital stays and frequent admissions for poor intake, vomiting, and dehydration, we have gradually modified our postoperative strategies with several preventative and therapeutic maneuvers. We have previously published our Enhanced Recovery After Surgery protocol for pancreas transplant recipients, which focuses largely on the management of GP and motility issues common among postoperative patients with longstanding diabetes.^31^ Nasogastric tubes (NGTs) are removed at the time of extubation, and a liquid diet is permitted during the early postoperative period. Currently, all recipients are initially started on scheduled metoclopramide (10 mg IV every 6 h) and then transitioned to oral administration before meals and at bedtime once tolerating a diet, unless contraindicated. This medication is rapidly tapered once the patients are discharged. Before incision at the time of transplant, all recipients undergo ultrasound-guided transversus abdominus plane (TAP) catheter placement or, more recently, injection with liposomal bupivacaine to minimize narcotic requirements during the postoperative stage.^32^ Methylnaltrexone (12 mg SC daily) is a peripheral-acting Mu-opioid receptor antagonist that has been added to the postoperative regimen, as well, to minimize narcotic impact on colon motility. In cases where gastric motility appears severely restricted or for repeat admissions for vomiting, mechanical obstruction is first ruled out with an oral contrast abdominal and pelvic computed tomography scan or, if inconclusive, computed tomography enteroclysis.^33,34^ Erythromycin is occasionally added to the regimen, but due to the known interaction with calcineurin inhibitors, this is seldom preferred. Esophagogastroscopy with intrapyloric botulinum toxin injection is also an option that can have a dramatic impact on gastric emptying in some patients. Finally, if recipients require repeat pylorus botulinum toxin injections, they are referred for G-POEM.^35^ Surgeries such as open or laparoscopic pyloroplasty/pyloromyotomy, gastrojejunostomy, or subtotal gastrectomy were extremely rarely offered or performed as a last resort after other interventions failed and only in the earliest periods of this cohort.

## RESULTS

There were 156 of 731 pancreas transplant recipients (21%) who were diagnosed pretransplantation with GP or had a history of GP-like symptoms (Table 1). The patients with GP were similar to patients without a diagnosis of GP with regard to type of pancreas transplant, race, and BMI. The patients with GP were significantly more likely to be female individuals (59% versus 40%, *P* < 0.001) and were younger (median age 41 versus 44 y, *P* < 0.01). Donor demographics were similar except for lower median BMI for the recipients with GP (*P* = 0.02). Posttransplant, there was no difference between patients with and without GP for length of hospital stay and for 90-d, 1-y, and 5-y patient survival or pancreas or renal (for SPK) allograft survival (Table 2). The patients with GP were more likely to be readmitted in the first 3 mo after transplant (61% versus 52%, *P* = 0.04) and to be specifically admitted for nausea and emesis (30% versus 15%, *P *= 0.04). Readmissions for nausea with emesis during the first posttransplant year were even more dramatically higher for patients with GP (38% versus 13%, *P* < 0.001). As a separate analysis, the groups were reanalyzed by transplant type as shown in Table 3. Most findings remained similar to the prior analysis with the exception of a significantly lower 90-d (but not 1 y) pancreas allograft survival (89% versus 97%, *P* = 0.04), patient survival (92% versus 100%, *P* < 0.001), and median KM patient survival (181 versus 214, *P* = 0.03) for recipients of PTA.

**TABLE 1.** Demographic data for 731 consecutive pancreas transplant recipients between 2003 and 2023

	n (%)	No gastroparesis	Gastroparesis	*P*
Overall	731 (100%)	575 (79%)	156 (21%)	
Recipient				
Transplant type				
SPK	454 (62%)	357 (62%)	97 (62%)	1.00
PAK	104 (14%)	82 (14%)	22 (14%)	
PTA	173 (24%)	136 (24%)	37 (24%)	
Sex				
Male	408 (56%)	345 (60%)	63 (41%)	<0.001
Female	318 (44%)	227 (40%)	91 (59%)	
Race				
White	650 (90%)	518 (91%)	132 (86%)	0.12
Black	64 (9%)	44 (8%)	20 (13%)	
Other	10 (1%)	8 (1%)	2 (1%)	
Age, y				
Median (SE)	43 (0.4)	44 (0.4)	41 (0.7)	<0.01
Body mass index				
Median (SE)	25.2 (0.2)	25.4 (0.2)	24.6 (0.4)	0.3
Donor				
Age, y				
Median (SE)	24 (0.4)	24 (0.4)	21 (0.9)	0.25
Body mass index				
Median (SE)	23.7 (0.2)	23.9 (0.2)	22.9 (0.3)	0.02
Transplant data				
Total ischemia time, h				
Median (SE)	7.4 (0.2)	7.7 (0.2)	7.5 (0.5)	0.56

PAK, pancreas transplant after previous kidney transplant; PTA, pancreas transplant alone; SPK, simultaneous pancreas and kidney.

**TABLE 2.** Clinical outcome data for 731 consecutive pancreas transplant recipients between 2003 and 2023

	n (%)	No gastroparesis	Gastroparesis	*P*
Overall	731 (100%)	575 (79%)	156 (21%)	
Length of hospital stay, d				
Mean, median (SE)	11.0, 7 (0.6)	10.7, 7 (0.6)	12.2, 8 (1.2)	0.28
Acute cellular rejection of pancreas within 1 y	4%	5%	3%	0.45
Any posttransplant readmission				
Within 3 mo	54%	52%	61%	0.04
Any readmission for nausea/emesis				
Within 1 y	18%	13%	39%	<0.001
Days to first readmission (only for readmissions)				
Mean, median (SE)	220, 29 (31)	234, 23 (41)	193, 32 (47)	0.53
No. of readmissions in 1 y posttransplant				
Mean, median (SE)	2.1, 1 (0.2)	1.8, 1 (0.2)	2.7, 1 (0.4)	0.02
Any endoscopic gastroduodenoscopy				
Pretransplant	5%	3%	10%	<0.001
Within 1 y posttransplant	19%	14%	39%	<0.001
After 1 y posttransplant	30%	27%	42%	<0.01
Graft survival (pancreas)				
90 d	95%	96%	94%	0.52
1 y	93%	93%	94%	0.87
Median survival Kaplan-Meier, mo	167	170	155	0.17
Patient survival				
90 d	98%	99%	97%	0.31
1 y	97%	97%	97%	0.78
Median survival Kaplan-Meier, mo	199	200	192	0.50
Graft survival (kidney for SPK)				
90 d	97%	97%	99%	0.26
1 y	96%	95%	99%	0.09
Median survival Kaplan-Meier, mo	172	175	159	0.31
Medication usage				
Pretransplant on admission				
Metoclopramide	12%	8%	29%	<0.001
Erythromycin	1%	<1%	1%	0.50
Odansetron	4%	2%	12%	<0.001
Promethazine	7%	4%	25%	<0.01
Domperadone	0%	0%	0%	NA
Posttransplant on discharge				
Metoclopramide	72%	72%	71%	0.89
Erythromycin	2%	1%	5%	<0.01
Odansetron	8%	5%	17%	<0.001
Promethazine	7%	4%	25%	<0.01
Domperadone	<1%	<1%	<1%	0.38
Gastric electric stimulator				
Pretransplant	2%	0%	8%	<0.001
Botulinum toxin injection of pylorus				
Pretransplant	2%	0%	11%	0.02
Any time posttransplant	22%	13%	44%	<0.001
Nutrition				
Serum albumin, mean, median (SE)				
At transplant	3.5, 3.5 (0.02)	3.5, 3.5 (0.03)	3.5, 3.5 (0.05)	0.41
1 y posttransplant	3.8, 3.8 (0.02)	3.7, 3.8 (0.02)	3.8, 3.8 (0.05)	0.72
Patient weight, mean, median (SE)				
Change from transplant to 1 y posttransplant, kg	1.0, 0.1 (0.5)	1.2, 0.2 (0.5)	0.2, –0.1 (1.0)	0.4
Change from transplant to 1 y posttransplant (%)	2%, 0.1% (0.6)	2%, 0.3% (0.7)	0.7%, –0.1% (1.4)	0.28

SPK, simultaneous pancreas and kidney.

**TABLE 3.** Clinical outcomes data for 731 consecutive pancreas transplant recipients between 2003 and 2023

		SPK				PAK				PTA			
	n (%)	Overall SPK	No gastroparesis	Gastroparesis	*P*	Overall PAK	No gastroparesis	Gastroparesis	*P*	Overall PTA	No gastroparesis	Gastroparesis	*P*
Overall	731 (100%)	n = 454	357 (79%)	91 (21%)		n = 104	82 (79%)	22 (21%)		n = 173	136 (79%)	37 (21%)	
Length of hospital stay, d													
Mean, median (SE)	11.0, 7 (0.6)	11.4, 8 (0.7)	10.7, 7 (0.7)	13.7, 9 (1.8)	0.08	12.1, 7 (2.3)	12.7, 7 (2.8)	10.3, 7 (2.4)	0.67	9.5, 7 (0.8)	9.5, 7 (0.9)	9.4, 7 (1.0)	0.92
Acute cellular rejection of pancreas within 1 y	4%	4%	4%	4%	0.94	1%	1%	0%	1.00	6%	7%	3%	0.34
Any posttransplant readmission													
Within 3 mo	54%	57%	54%	67%	0.02	46%	45%	50%	0.68	50%	50%	51%	1%
Any readmission for nausea/emesis													
Within 1 y	18%	15%	9%	38%	<0.001	15%	15%	18%	0.68	27%	21%	51%	<0.001
Days to first readmission (only for readmissions)													
Mean, median (SE)	220, 29 (31)	184, 21 (35)	174, 16 (41)	200, 32 (65)	0.72	360, 43 (167)	366, 43 (208)	338, 310 (190)	0.95	272, 68 (62)	341, 83 (92)	151, 33 (49)	0.14
No. of readmissions in 1 y posttransplant													
Mean, median (SE)	2.1, 1 (0.2)	1.8, 1 (0.1)	1.6, 1 (0.2)	2.2, 1 (0.3)	0.07	1.3, 1 (0.3)	1.3, 1 (0.3)	1.4, 1 (0.5)	0.90	3.2, 2 (0.5)	2.7, 2 (0.5)	4.2, 2 (1.2)	0.16
Any endoscopic gastroduodenoscopy													
Pretransplant	5%	5%	3%	9%	0.01	5%	2%	14%	0.06	5%	4%	11%	0.10
Within 1 y posttransplant	19%	18%	12%	39%	<0.001	10%	5%	27%	<0.01	28%	24%	46%	0.01
After 1 y posttransplant	30%	22%	18%	37%	<0.001	37%	33%	50%	0.14	48%	48%	49%	0.86
Graft survival													
90 d	95%	95%	96%	95%	0.78	94%	93%	100%	0.34	95%	97%	89%	0.04
1 y	93%	94%	94%	95%	0.78	92%	92%	96%	0.53	91%	92%	89%	0.60
Median survival Kaplan-Meier, mo	167	178	180	167	0.40	150	150	147	0.76	154	151	133	0.24
Patient survival													
90 d	98%	99%	98%	99%	0.65	98%	98%	100%	1.00	98%	100%	92%	<0.01
1 y	97%	97%	97%	99%	0.26	96%	96%	96%	1.00	97%	99%	92%	0.07
Median survival Kaplan-Meier, mo	199	197	198	191	0.85	184	180	196	0.31	211	214	181	0.03
Medication usage													
Pretransplant on admission													
Metoclopramide	12%	13%	8%	31%	<0.001	20%	17%	31%	0.19	6%	2%	21%	<0.001
Erythromycin	1%	1%	1%	1%	0.50	0%	0%	0%	NA	0%	0%	0%	NA
Odansetron	4%	3%	1%	13%	<0.001	0%	0%	0%	NA	8%	6%	14%	0.16
Promethazine	7%	8%	5%	28%	<0.01	0%	0%	0%	NA	0%	0%	0%	NA
Domperadone	0%	0%	0%	0%	NA	0%	0%	0%	NA	0%	0%	0%	NA
Posttransplant on discharge													
Metoclopramide	72%	74%	75%	71%	0.41	59%	60%	57%	0.83	73%	71%	81%	0.25
Erythromycin	2%	2%	1%	6%	<0.01	0%	0%	0%	NA	2%	2%	3%	0.51
Odansetron	8%	7%	3%	18%	<0.001	4%	4%	5%	0.82	12%	10%	22%	0.04
Promethazine	7%	8%	5%	28%	<0.01	0%	0%	0%	NA	0%	0%	0%	NA
Domperadone	<1%	<1%	<1%	1%	0.39	0%	0%	0%	NA	0%	0%	0%	NA
Gastric electric stimulator													
Pretransplant	2%	2%	0%	9%	<0.001	1%	0%	5%	0.21	2%	0%	8%	<0.01
Botulinum toxin injection of pylorus													
Pretransplant	2%	2%	0%	12%	0.02	0%	0%	0%	NA	0%	0%	0%	NA
Any time posttransplant	22%	20%	11%	42%	<0.001	0%	0%	0%	NA	33%	22%	58%	0.04
Nutrition													
Serum albumin, mean, median (SE)													
At transplant	3.5, 3.5 (0.02)	3.4, 3.4 (0.02)	3.5, 3.4 (0.03)	3.4, 3.9 (0.04)	0.70	3.6, 3.6 (0.05)	3.6, 3.7 (0.06)	3.6, 3.6 (0.1)	0.82	3.5, 3.6 (0.05)	3.6, 3.6 (0.05)	3.5, 3.5 (0.1)	0.39
1 y posttransplant	3.8, 3.8 (0.02)	3.8, 3.9 (0.02)	3.8, 3.9 (0.03)	3.8, 3.9 (0.04)	0.76	3.7, 3.7 (0.04)	3.7, 3.7 (0.05)	3.8, 3.8 (0.1)	0.19	3.7, 3.8 (0.04)	3.7, 3.8 (0.04)	3.5, 3.8 (0.1)	0.07
Patient weight, mean, median (SE)													
Change from transplant to 1 y posttransplant, kg	1.0, 0.1 (0.5)	2.6, 1.5 (0.6)	2.6, 1.7 (0.6)	2.4, 0.5 (1.3)	0.89	0.4, 0 (1.2)	0.5, 0 (1.5)	–0.05, 0.05 (1.5)	0.84	–2.8, 2.9 (1.0)	–2.1, 2.2 (1.1)	–5.7, –5.1 (2.0)	0.15
Change from transplant to 1 y posttransplant (%)	2%, 0.1% (0.6)	4%, 2% (0.8)	4%, 2.5% (0.9)	4%, 0.5% (1.9)	0.83	2%, 0% (1.9)	2%, 0% (2.3)	<1%, <1% (2.2)	0.73	–3%, –4% (1.2)	–2%, –3% (1.4)	–7%, –7% (2.3)	0.06

PAK, pancreas transplant after previous kidney transplant; PTA, pancreas transplant alone; SPK, simultaneous pancreas and kidney.

Uses of promotility agents are also summarized in Table 2. There was a dramatic increase in the use of promotility agents after transplant related to the posttransplant Enhanced Recovery After Surgery protocol, but these medications were weaned in the majority of patients in the postoperative period. There was no difference in the incidence of acute cellular rejection within 1 y of transplant. Baseline and 1-y serum albumin levels were similar between the groups, and there was no significant difference in ΔBMI at 1 y between groups. Patients with GP were more likely to be on metoclopramide, ondansetron, and promethazine pretransplant and were more likely to remain on promethazine or have erythromycin added than patients without GP. Requirement for endoscopic botulinum toxin injection of the pylorus was more common in patients with GP. Pretransplant requirement for pyloric botulinum toxin injection was exclusively observed in recipients with a diagnosis of GP before transplant and was required more frequently posttransplant in patients with a pretransplant diagnosis of GP (44% versus 13%, *P* < 0.001). The fact that there were individuals who did not have a diagnosis of GP before transplant but who ultimately required intervention after transplant highlights the fact that all recipients, including those without a diagnosis of GP before transplant, are still at risk of having diabetes-related motility issues after transplant. Insertion of gastric stimulators and G-POEM procedures were exclusively performed in recipients with GP. Additionally, we also had 1 recipient who underwent extensive surgery, including pyloroplasty, gastrojejunostomy, gastric stimulator, and ultimately near total gastrectomy as well as several lysis of adhesions and incisional hernia repairs. Overall, this patient has had >20 abdominal surgeries during the span of the 15 y since her PTA. Despite all of these interventions, this patient continued to have severe symptoms from dysmotility that never resolved.

**FIGURE 1.**
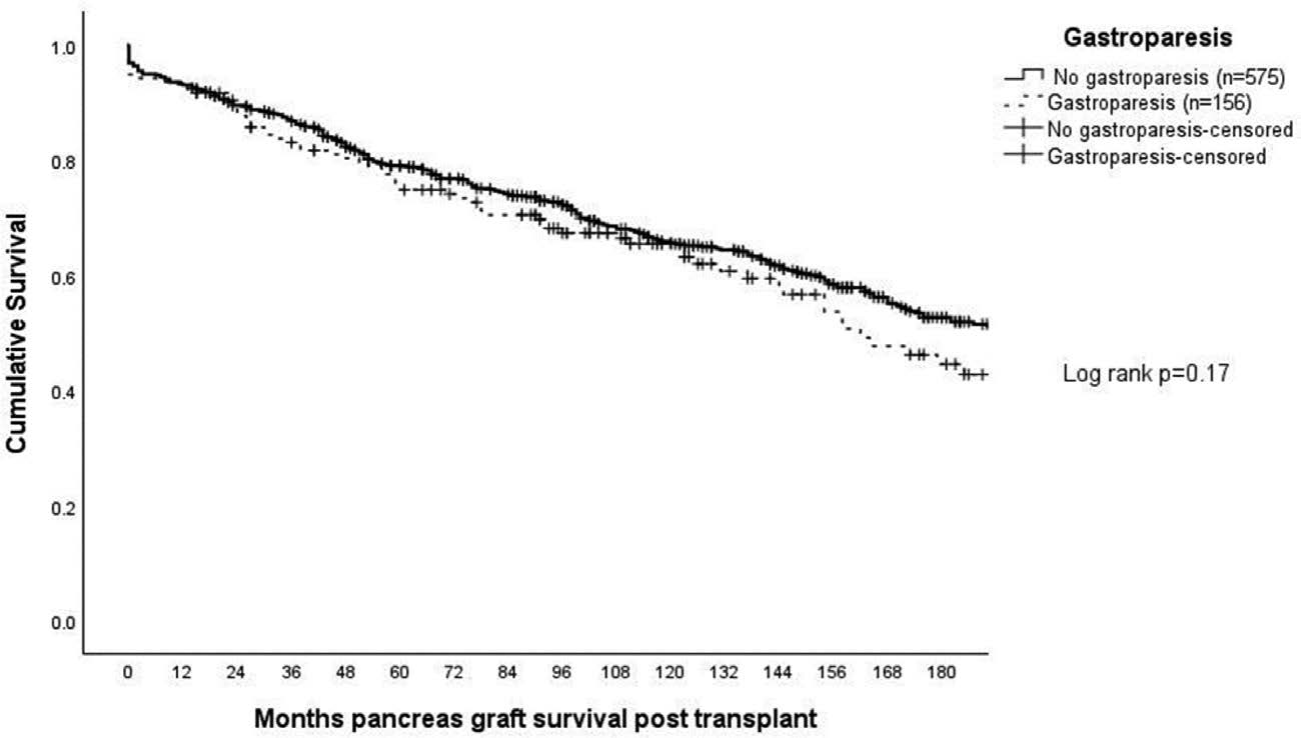
Kaplan-Meier analysis of pancreas allograft survival up to 15 y posttransplant comparing those with and without a diagnosis of gastroparesis before pancreas transplantation (n = 731).

**FIGURE 2.**
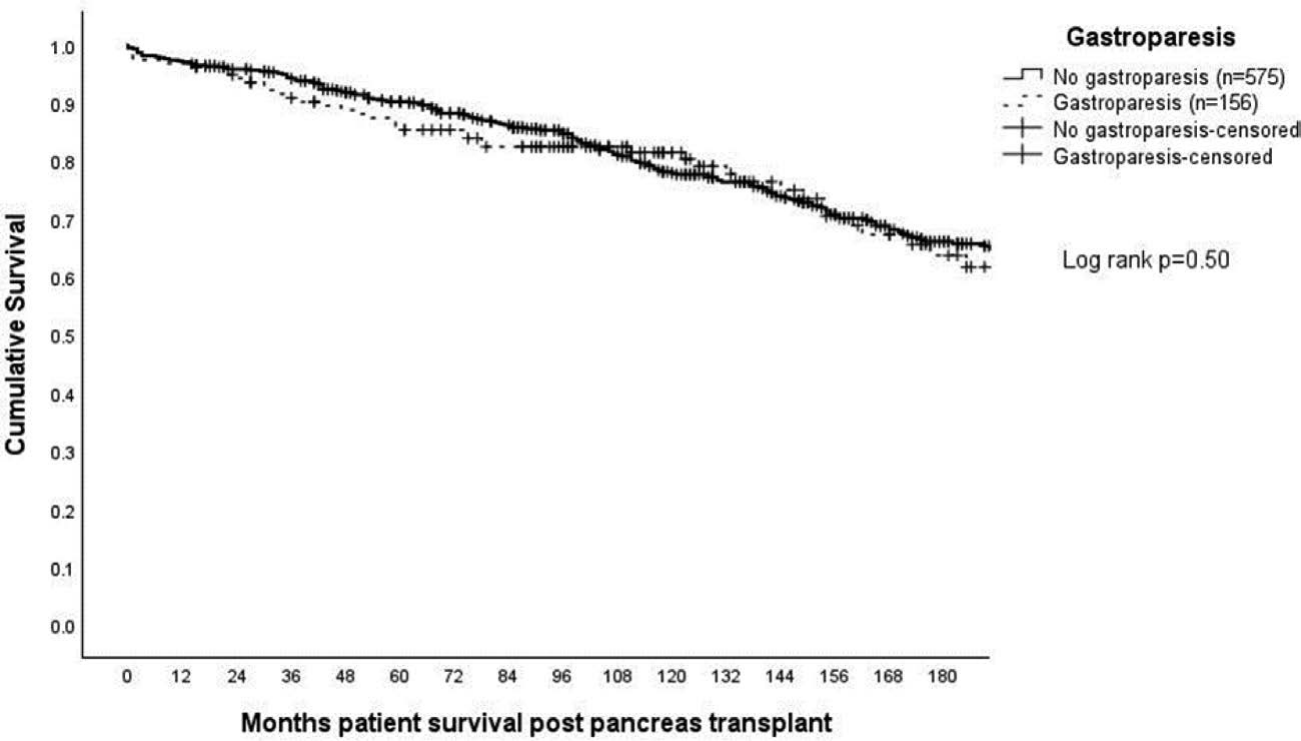
Kaplan-Meier analysis of patient survival up to 15 y posttransplant comparing those with and without a diagnosis of gastroparesis before pancreas transplantation (n = 731).

Kaplan-Meier analysis of pancreas allograft and patient survival are shown in Figures 1 and 2 and demonstrated similar survival between the patients with and without GP up to 15 y posttransplant (*P* = 0.17 and *P* = 0.5, respectively). Kaplan-Meier analysis of kidney allograft survival for SPK recipients is included in Table 2 and was also similar for patients with and without GP up to 15 y posttransplant (*P* = 0.31). There were a total of 46 deaths at any point after transplant among the 156 patients with gastroparesis (29%), with 26 of these deaths occurring >5 y and 19 of those >10 y after transplant. These deaths were reviewed in detail. Only 4 of these deaths occurred within the first 3 mo after surgery. One of these deaths was the result of aspiration at the time of endoscopy for an upper gastrointestinal bleed. The other 3 early deaths were not related to the presence of GP at all.

## DISCUSSION

Gastroparesis is a common complication of diabetes and therefore will be encountered with some frequency in the pancreas transplant patient population. To date, there have been very few reports addressing the impact of pancreas transplantation on gastroparesis and the impact of gastroparesis on the outcome of pancreas transplantation.

Currently, there are very few management options available for this disorder. There is no ideal medication: metoclopramide is effective, but it is not ideal because of the potential for extrapyramidal side effects and the potential development of tachyphylaxis. Erythromycin is also very effective, but it interacts with immunosuppression medications, particularly calcineurin inhibitors, such as tacrolimus and cyclosporine, and mammalian target of rapamycin inhibitors, such as sirolimus. Furthermore, long-term usage of erythromycin may be associated with the development of tachyphylaxis. Other medications, such as domperidone and cisapride, for example, exist but are not approved in the United States by the US Food and Drug Administration. More invasive strategies include endoscopic intrapyloric botulinum toxin injection, which works in select cases, and gastric stimulators, which would require relaparotomy or laparoscopy for insertion and also only works in select cases. The use of endoscopic botulinum toxin injection for treating severe gastroparesis post–pancreas transplantation has been previously described.^14,15^ Both studies describe 3 patients each, one of which was treated while awaiting islet cell transplantation. As in their experience, this typically is effective but not sustained. This procedure can be repeated after 3–6 mo if symptoms recur. With controlled diabetes provided by pancreas transplantation, it is unusual to have persistent GP symptoms after this time interval, but we do have the occasional recipient that requires this intervention every 3–6 mo long-term with excellent symptom control. More recently, we have offered recipients that require repeat botulinum toxin injections G-POEM as a more effective long-term solution.^35^ We have transplanted patients with implanted gastric stimulators, which have been used for the control of GP symptoms. These devices help with nausea but do not actually improve motility. We have rarely used this treatment modality postoperatively because in many cases GP improves with time after pancreas transplantation, and it would be preferable to avoid the second operation and the foreign body. Laparoscopic placement of this type of device post–pancreas transplantation loss has however been described with the dual goal of managing symptoms of GP and achieving weight with good results.^36^ As mentioned in the results section, we did have 1 recipient who underwent extensive surgical intervention for gastroparesis and bowel dysmotility. The literature suggests that procedures such as pyloroplasty, gastrojejunostomy, and gastrectomy are last-resort treatments reserved for select patients after extensive evaluation and exhaustion of other options.^37,38^ The results are usually disappointing and rarely resolve the symptoms, as was the case with our recipient.

Our strategy developed in stages throughout the entire study period. Currently, we use metoclopramide (transitioned from intravenous to oral administration and then gradually weaned) in the early postoperative period, early resumption of diet, narcotic minimization strategies including placement of TAP catheters and blocks and subcutaneous methylnaltrexone to counteract the colonic motility delay associated with narcotic use. It has been our impression that we have been making progress with this disorder, as is supported by the fact that length of stay and survival are similar to those of patients without pretransplant diagnosis of GP. It is our opinion that stimulating the gastrointestinal tract rather than fasting the patient for several days is one of the key elements to bowel recovery. As was reported by the University of Wisconsin, we have found that removal of NGTs at the time of endotracheal extubation and early introduction of diet was actually well tolerated, with very few patients requiring replacement of the NGT.^39^ In fact, for patients that are struggling with poor intake secondary to nausea as a symptom of gastroparesis, we have found that appetite stimulants such as dronabinol, megestrol acetate, and cyproheptadine may be very helpful. A brief course of enteral feeds through a nasoduodenal or nasojejunal feeding tube may also break the cycle of anorexia, poor intake, and nausea, as fasting can lead to atrophy of the bowel mucosa, so stimulation and delivery of essential amino acids may be paramount to achieving recovery. Additionally, these feeding tubes traverse the pylorus, effectively stenting the gastric outlet, thereby improving gastric emptying mechanically. Minimizing narcotic usage is also essential. It is our strongly held opinion that it is inappropriate to manage the symptoms of nausea, distension, bloating, and constipation common in the GP patient population with narcotics, as this only makes the situation worse. Postoperatively, it is difficult to avoid narcotics entirely. To minimize utilization, nonnarcotic strategies, including local anesthesia delivered via TAP catheters or long-lasting TAP injections, provide sustained but not complete relief. Tramadol is a nonnarcotic analgesic that has less constipating tendencies than oral narcotics. Rarely, ketorolac can be added but only under close observation as generally nonsteroidal anti-inflammatories are relatively contraindicated in the renal transplant recipient population. Methylnaltrexone is a peripherally acting µ-opioid antagonist that acts to reverse some of the side effects of opioid medications, such as constipation, without affecting analgesia. Alternatives include alvimopan and naloxegol.

One of the goals of this study was to determine whether GP, as diagnosed on clinical history or scintigraphy, had a negative impact on outcomes after pancreas transplantation. It is likely that there were many recipients with subclinical delayed gastric emptying or gastrointestinal motility issues, which is why the posttransplant recovery pathway is applied to all patients, regardless of gastric-emptying study results. The outcomes studied suggest that the presence of clinically evident GP pretransplant did not have a significant impact on length of stay or pancreas allograft and patient survival, although readmissions were significantly more common. These results suggest that the presence of GP should not be considered a contraindication to pancreas transplantation.

## CONCLUSIONS

GP is identified with increased frequency among the specific patient population referred for pancreas transplant and, although it does not seem to affect allograft or patient survival, does seem to have an impact on readmissions and the need for interventions. Strategies to manage GP immediately after transplant can be very helpful in improving symptoms during the postoperative period and preventing readmission for this patient population.
